# Functionalization of Stainless Steel with Hyperbranched Poly(viologen) Brushes for Enhanced Antimicrobial, Antifouling and Anticorrosion

**DOI:** 10.3390/molecules30112427

**Published:** 2025-05-31

**Authors:** Huaqiang He, Youquan Liu, Wei Yang, Siqi Liu, Jie Wang, Zicheng Peng, Shaojun Yuan

**Affiliations:** 1 Research Institute of Natural Gas Technology, PetroChina Southwest Oil & Gasfield Company, Chengdu 610299, China; hehuaqiang@petrochina.com.cn (H.H.); youquan_1@petrochina.com.cn (Y.L.); yang_wei001@petrochina.com.cn (W.Y.); 2 Research Institute of Safety, Environment Protection and Technology Supervision, PetroChina Southwest Oil & Gasfield Company, Chengdu 610299, China; liusiqi@petrochina.com.cn; 3 PetroChina Southwest Oil & Gas-Field Company, Chengdu 610051, China; wangjie16@petrochina.com.cn (J.W.); pengzch@petrochina.com.cn (Z.P.); 4 Low-Carbon Technology & Chemical Reaction Engineering Lab, College of Chemical Engineering, Sichuan University, Chengdu 610065, China

**Keywords:** stainless steel, hyperbranched polymer, antimicrobial, antifouling, biocorrosion

## Abstract

To enhance the resistance of stainless steel (SS) against biofouling and biocorrosion, hyperbranched poly(viologen) brushes were covalently immobilized onto SS substrates. This study systematically evaluated the efficacy of the functionalized SS substrates in inhibiting microorganism adhesion, biofouling and biocorrosion. Enhanced antifouling and antimicrobial properties were evidenced through assays involving the attachment of *Amphora coffeaeformis*, the settlement of *Pseudomonas* sp. bacteria and barnacle cyprids. Furthermore, the functionalized SS substrates demonstrated superior antifouling performance alongside excellent biocorrosion–inhibition properties. These findings suggest that the functionalized SS substrates, with their robust antimicrobial, antifouling and anticorrosion capabilities, hold significant potential for applications in aquatic environments

## 1. Introduction

Stainless steels (SS) have been extensively utilized in marine and freshwater environments because of their durability, high stability and excellent corrosion resistance, which is attributed to a thin, compact, chromium-enriched oxide layer. However, when exposed to marine environments, SS continues to face the persistent challenge of biofouling caused by various microorganisms [1,2]. Marine biofouling, characterized by the formation of biofilms due to the colonization of micro- and macro-marine organisms on submerged surfaces, poses significant challenges to shipping vessels and marine structures [3,4]. This phenomenon creates a corrosive environment that leads to pitting corrosion in metals, causing material degradation and structural failure [5]. SS is especially susceptible to biocorrosion, commonly known as microbiologically influenced corrosion (MIC). This form of corrosion is primarily driven by the activity of sulfate-reducing bacteria (SRB) and is exacerbated by localized corrosion resulting from the presence of chloride ions and reducing sulfur compounds. Biocorrosion has become a critical issue in marine and industrial environments, as it is responsible for approximately 20% of all corrosion, incurring an estimated global cost of US $30–50 billion per year to the maritime industry [6,7]. Among the various risks and concerns related to biofilm formation, biocorrosion is of particular industrial significance due to its potential to compromise structural integrity, resulting in technical failures [8,9,10].

Self-polishing paints containing biocides such as tributyltin, copper, zinc and others have been widely employed to control and manage marine biofouling [11,12]. However, the release of these biocides into the marine environment has severe adverse effects on marine organisms. Consequently, the development of non-leaching biocide and antifouling coatings has become increasingly desirable. Modifying material surfaces with non-toxic functional polymer brushes offers an environmentally benign approach to minimize and prevent marine biofouling without releasing harmful biocides [5,13].

Surface-initiated atom transfer radical polymerization (SI-ATRP) is a highly universal and efficient technique for the fabrication of dense and stable polymeric functional brushes. This technique allows for precise control over brush properties, enabling the creation of narrow polydispersity, controlled architecture and well-defined compositions [14,15,16,17,18]. Surface properties such as wettability and charge are critical in determining microfouling and macrofouling processes. For instance, crosslinked hydrogel brushes grafted onto SS surfaces via SI-ATRP have demonstrated effective antifouling performance in marine environments [19]. Quaternized poly(2-(dimethylamino)ethyl methacrylate) (P(DMAEMA)) brushes, which exhibit antibacterial activity through quaternary ammonium groups that disrupt bacterial cell membranes, have been immobilized on SS surfaces to inhibit biocorrosion [20,21]. Similarly, viologen moieties, known for their dual bactericidal and corrosion-inhibition properties, have been used to functionalize oxidized Cu–Ni alloy surfaces for combating MIC [22]. It is therefore anticipated that quaternized poly(viologen) brushes may demonstrate exceptional antifouling and anticorrosion capabilities.

**Scheme 1:**
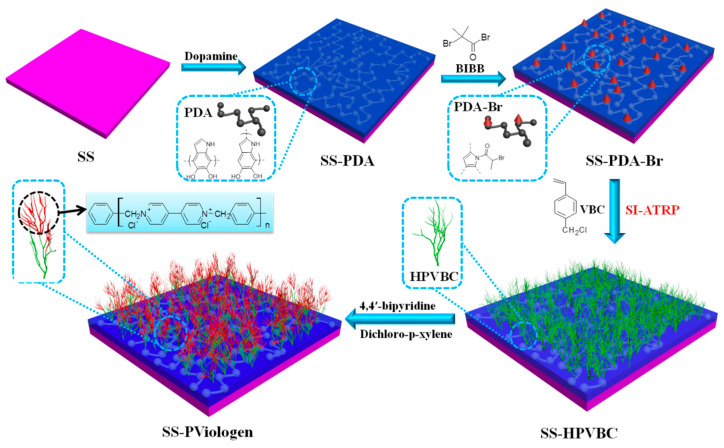
Schematic illustration of the preparation of hyperbranched poly(viologen)-functionalized SS surface.

In this study, hyperbranched poly(viologen) brushes were functionalized on SS surfaces to confer bactericidal, antifouling and corrosion-inhibition properties, aimed at mitigating biofouling and biocorrosion. As depicted in Scheme 1, a polymeric precursor, polydopamine (PDA), was first covalently immobilized onto oxidized original SS substrates to anchor an alkyl halide ATRP initiator. Subsequently, the surface-initiated ATRP of 4-vinylbenzyl chloride (VBC) was conducted from the SS-PDA-Br surface to create hyperbranched poly(vinyl benzyl chloride) polymer brush layers. Viologen moieties were introduced onto the metallic substrate surface through covalent interactions between the anchored benzyl chloride groups and bipyridine, facilitated by the addition of α,α′-dichloro-p-xylene to form hyperbranched viologen-containing polymer chains. The antibacterial, antifouling and corrosion-inhibition properties of the modified metallic surfaces were comprehensively evaluated through bacterial adhesion assays, *Amphora coffeaeformis* assays, barnacle cyprid settlement assays and electrochemical studies. The main novelties of this study are as follows: (i) Few studies have explored the use of hyperbranched PViologen containing quaternary ammonium groups as multifunctional coatings with both antifouling and anticorrosion properties. (ii) The synthesis of polycationic polymer chains by combining surface-initiated ATRP of PVBC with the covalent coupling of viologen has been scarcely reported. The quaternary ammonium groups on the SS-PViologen surface can be precisely controlled by adjusting the grafting density of PVBC and the amount of viologen and α,α′-dichloro-p-xylene. (iii) This study also comprehensively investigates the antifouling performance of PViologen-functionalized surfaces against three representative marine fouling organisms, such as marine bacteria (*Pseudomonas* sp.), microalgae (*Amphora coffeaeformis*) and macrofouling larvae (*Balanus Amphitrite*), which settle at various fouling stages, a topic rarely addressed in previous studies. *Pseudomonas* sp., a common Gram-negative bacterium in marine environments, was selected as a pioneer organism in biofilm formation. Inhibiting the adhesion of *Pseudomonas* can directly modulate the initial stage of biofouling. *Amphora coffeaeformis*, a widely distributed marine diatom (microalga), was chosen as an early colonizer in biofilm development, as it forms the basis for the subsequent attachment of larger fouling organisms, such as barnacles and mussels. *Balanus amphitrite*, commonly known as the striped barnacle, was used to represent macrofouling larvae. The adhesion of its larvae and the subsequent formation of calcified shells in adults are key challenges in marine biofouling. The attachment of barnacle larvae signifies a transitional phase in the fouling process, progressing from microbial biofilm colonization (e.g., bacteria and diatoms) to macrofouling by larger organisms.

## 2. Results and Discussion

### 2.1. Anchoring of Polydopamine and ATRP Initiator onto the Surface of SS Substrates

**Figure 1:**
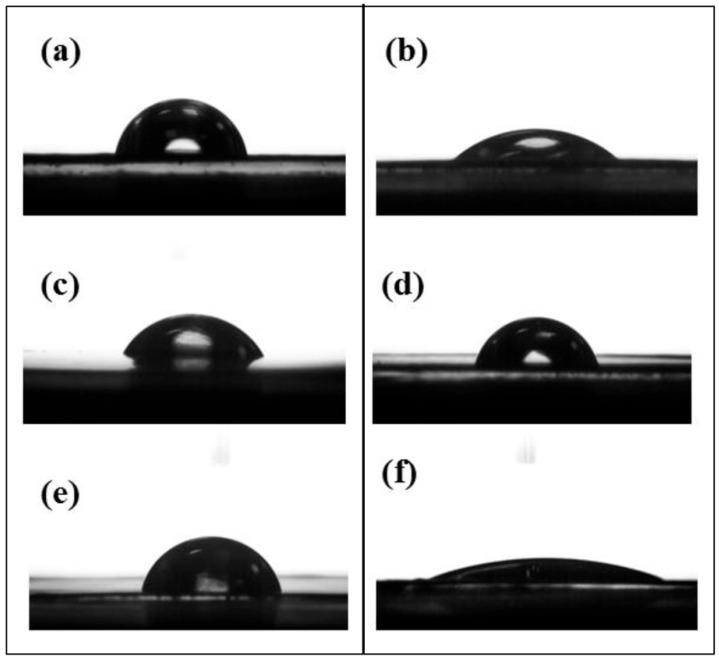
Images of a water droplet dropping on the surface of the (**a**) original SS, (**b**) SS-OH, (**c**) SS-PDA, (**d**) SS-PDA-Br, (**e**) SS-HPVBC and (**f**) SS-PViologen substrates.

Piranha solution, known for its strong oxidizing properties, has been widely recognized for its effectiveness in regenerating a fresh, oxidized and hydroxylated surface on various metals. The surface composition of the hydroxylated SS surface, referred to as the SS-OH surface after treatment with Piranha solution, was characterized using X-ray photoelectron spectroscopy (XPS). The presence of hydroxyl groups (-OH) significantly enhanced the hydrophilicity of the SS surface, consistent with a previous work [21]. This was evidenced by a decrease in the static water contact angle from approximately 77 ± 3° to 36 ± 2°, as presented in Table 1 and illustrated in Figure 1a,b.

Dopamine is a simple molecule that plays a critical role in both the formation of marine mussel adhesive and the mimicry of its proteins. It contains a catechol functional group, which serves as a universal bio-inspired site for attaching functional molecules and polymers to various surfaces [23,24,25,26]. In the present work, dopamine took place self-polymerization to form an adherent polydopamine (PDA) layer firmly anchoring on the surface of SS substrates (Scheme 1). While the exact polymerization mechanism remains unclear, the process likely involves the oxidation of the catechol group in dopamine to quinone, followed by rearrangement into dihydroxyindole, leaving abundant -OH and amine (-NH_2_) groups on the surface. The PDA-immobilized SS surface offers abundant -OH and -NH_2_ groups that can react with 2-bromoisobutyryl bromide, facilitating the introduction of an alkyl halide ATRP initiator. This functionalization facilitates subsequent SI-ATRP, allowing the grafting of linear or cross-linked polymer brushes onto the SS substrates.

**Figure 2:**
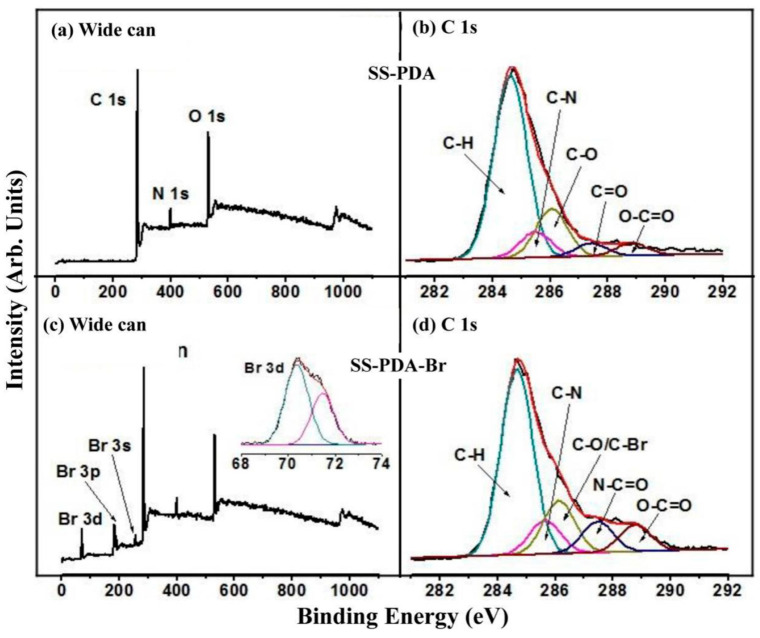
The (**a**) survey scan and (**b**) high-resolution C 1s core XPS spectra of the PDA-coated SS (SS-PDA) substrate surface, and the (**c**) survey scan and (**d**) high-resolution C 1s core spectra of the BIBB-immobilized SS (SS-PDA-Br) surface (inset is the high-resolution Br 3d core spectrum).

The successful anchoring of PDA onto the SS substrates was ascertained through XPS characterization. The high-resolution C 1s core spectrum (Figure 2b) of the PDA-functionalized SS surface (designated as SS-PDA) was deconvoluted into five peak components with binding energies (BEs) at approximately 288.4, 287.5, 286.2, 285.5 and 284.6 eV, corresponding to O–C=O, C=O, C–O-C, C–N and C–H species [19], respectively. The presence of the C–N peak component at a BE of 285.5 eV in the deconvoluted C 1s spectrum, along with the N 1s peak at approximately 400 eV observed in the wide-scan spectrum of SS-PDA (Figure 2a), further corroborated the existence of a PDA layer on the surface. The nitrogen-to-carbon ([N]/[C]) ratio of the PDA-coated SS substrate surface was measured at 0.11, which closely aligns with the theoretical value of 0.12 for dopamine [19]. Additionally, the SS-PDA surface exhibited increased hydrophobicity, as evidenced by the rise in the static water contact angle (WCA) to 60 ± 3° (Figure 1c, Table 1), further confirming the successful anchoring of the PDA layer on the SS-OH substrate.

The SS-PDA substrate offers abundant -OH and -NH_2_ functional groups, enabling reactions with BIBB to immobilize the ATRP initiator. The wide-scan spectrum of the ATRP initiator-immobilized surface (SS-PDA-Br) was shown in Figure 2c. The emergence of three new peaks at BEs of approximately 256, 189 and 70 eV, corresponding to Br 3s, Br 3p and Br 3d photoelectron signals, respectively, confirmed the successful incorporation of alkyl bromide-containing ATRP initiators onto the SS-PDA surface. The characteristic Br 3s, 3p and 3d spectra provide additional evidence of covalently bonded alkyl bromide species on the SS-PDA-Br surface. The static WCA value increased slightly from 60 ± 3° to 68 ± 2° for the SS-PDA-Br surface (Table 1, Figure 1c,d). The C 1s core-level spectrum of the SS-PDA-Br surface (Figure 2d) was deconvoluted into five peak components with BEs at 288.9, 287.5, 286.2, 285.6 and 284.6 eV, corresponding to O–C=O, N–C=O, C–O/C–Br, C–N and C–H species [19], respectively. Additionally, the high-resolution Br 3d core spectrum of the SS-PDA-Br surface (inset of Figure 2c) was deconvoluted into two peaks at BEs of 70.4 and 71.5 eV, corresponding to the spin–orbital splitting doublet of Br 3d_5_/_2_ and Br 3d_3_/_2_ species [19], respectively. These observations confirm the successful covalent attachment of alkyl bromide species to the SS-PDA surface.

### 2.2. Surface-Initiated ATRP of Vinyl-Benzyl Chloride

The SS-PDA-Br surface provided alkyl halide initiating sites for the preparation of well-defined polymer brushes via SI-ATRP. In the reaction mixture, 4-vinylbenzyl chloride inimer molecules functioned simultaneously as free initiators at the beginning of the ATRP process and as “branching units” in the synthesis of hyperbranched polymer brushes. To synthesize the SS-HPVBC surfaces, the molar ratio of [VBC (inimer)]:[CuBr (catalyst)]:[PMDETA (ligand)] was controlled at 100:1:1. The presence of grafted hyperbranched poly(vinylbenzyl chloride) (HPVBC) brushes on the SS-PDA-Br surface was confirmed through XPS analyses and static water contact angle measurements.

**Figure 3:**
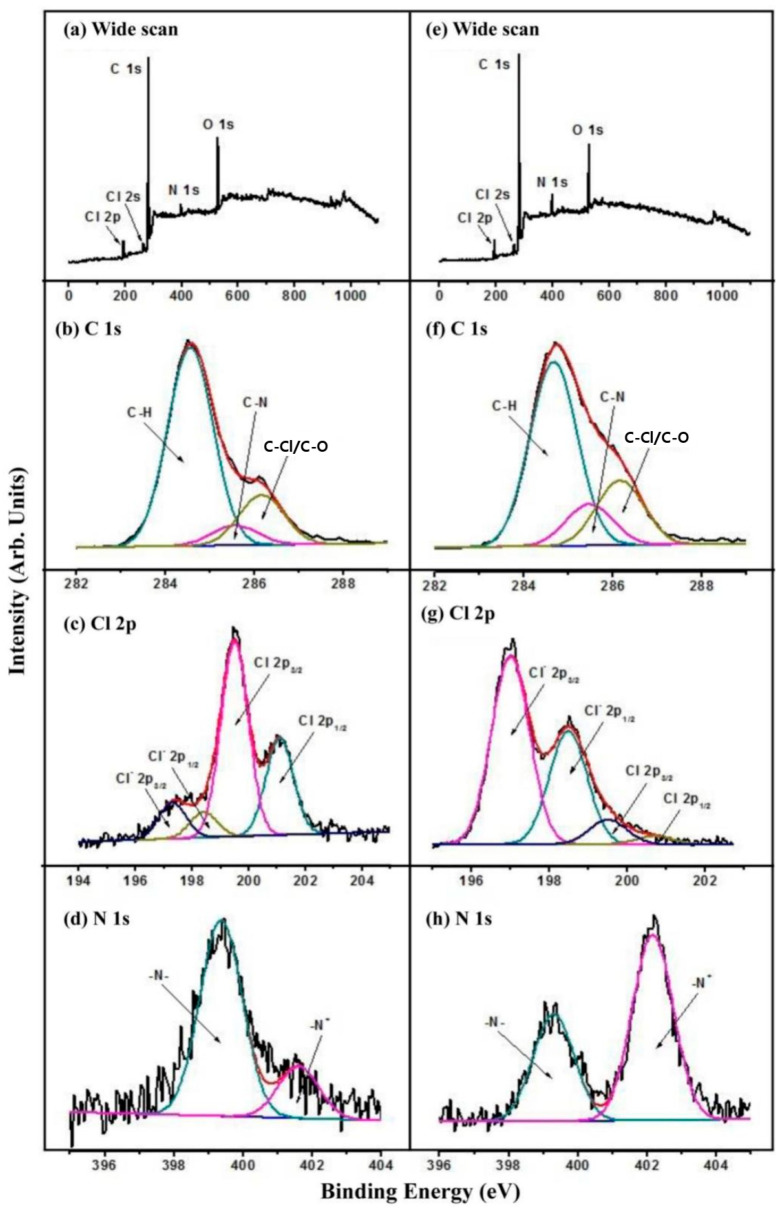
The (**a**) survey scan, (**b**) C 1s, (**c**) Cl 2p and (**d**) N 1s high-resolution core XPS spectra of the SS-HPVBC surface, and (**e**) survey scan, (**f**) C 1s, (**g**) Cl 2p and (**h**) N 1s high-resolution core XPS spectra of the SS-PViologen surface.

After the grafting of HPVBC onto the SS-PDA-Br surface, the functionalized surface demonstrated enhanced hydrophobicity, as evidenced by an increase in the static WCA to 73 ± 2° (Table 1 and Figure 1e). The survey scan XPS spectra (Figure 3a) of the HPVBC-grafted SS surface (referred to as SS-HPVBC), revealed the disappearance of the Br signal and the emergence of a peak at a BE of approximately 200 eV, indicative of Cl 2p photoelectron signals, confirmed the presence of covalently bonded chloride species derived from the benzyl chloride moiety of the grafted VBC units in the HPVBC chains. This result indicated that a substantial and dense layer of HPVBC brushes had been grafted onto the surface of the SS-PDA-Br substrate. The high-resolution C 1s core XPS spectrum (Figure 3b) of the SS-HPVBC surface was deconvoluted into three peak components with BEs of approximately 286.2, 285.5 and 284.6 eV, corresponding to C-Cl/C-O, C-N and C-H species [20], respectively. The presence of C–Cl peak component in the deconvoluted C 1s core-level XPS spectrum confirmed the successful grafting of the HPVBC brush layer onto the SS-PDA-Br surface. Additionally, the curve-fitted Cl 2p high-resolution core XPS spectrum (Figure 3b) exhibited spin–orbital splitting doublets of Cl 2p_3_/_2_ and Cl 2p_1_/_2_ peaks at BEs of approximately 200.1 and 202.2 eV [22], respectively, further verifying the presence of covalently bonded benzyl chloride species.

### 2.3. Coupling of Viologen on the SS-HPVBC Surface

Viologen moieties were introduced onto the SS-HPVBC surface through its reaction with an equimolar mixture of α,α′-dichloro-p-xylene and 4,4′-bipyridine [22]. The terminal pyridine groups on the SS-PViologen surface were subsequently quaternized with benzyl chloride and dichloro-p-xylene, forming viologen moieties on the SS surface. The XPS survey scan XPS spectra of the SS-HPVBC (Figure 3a) and SS-PViologen (Figure 3e) surfaces showed no significant differences, apart from minor variations in the relative intensities of C, Cl and N signals. The C 1s core-level spectra of SS-PViologen (Figure 3f) and SS-HPVBC (Figure 3b) surfaces were similar, except for an increased intensity of the peak component at a BE of 285.5 eV, corresponding to C-N species [22]. This increase indicated a high degree of immobilization of pyridine groups. The corresponding N 1s core-level spectrum (Figure 3h) displayed a predominant peak at a higher BE of 401.8 eV, attributed to the positively charged nitrogen (N^+^) in the quaternized viologen molecules. Based on the [N^+^]/[N_t_] ratio, the degree of quaternization of the pyridine rings was determined to be approximately 63.43%. The Cl 2p core-level spectrum of the SS-PViologen surface (Figure 3g) revealed two spin–orbit doublets, corresponding to ionic chlorine (Cl^−^) and covalent chlorine (-Cl) species. The high ratio of [Cl^−^]/[Cl_t_], approximately 88.9%, suggested that the incorporated pyridine groups reacted extensively with benzyl chloride, resulting in the formation of a viologen-functionalized surface. The presence of a small amount of covalent chlorine (-Cl) was attributed to residual benzyl chloride that had not fully reacted with bipyridine. Due to the hydrophilic nature of the viologen moieties, the viologen-functionalized SS surface (SS-PViologen) exhibited a significantly reduced static water contact angle of 17 ± 1° (Table 1, Figure 1f), indicating a highly hydrophilic surface.

### 2.4. Evaluation of Antimicrobial and Anti-Adhesion Properties of the Functionalized SS Surfaces

**Figure 4:**
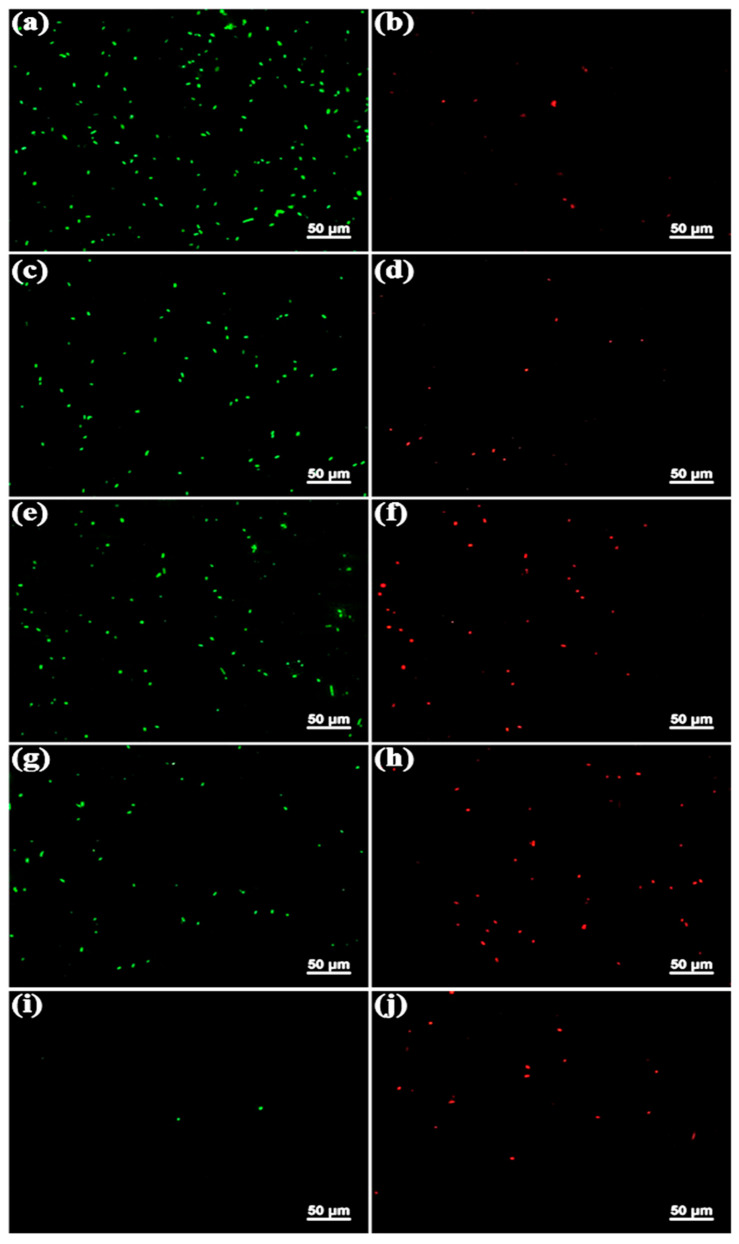
Fluorescence micrographs (1000×) of the (**a**,**c**,**e**,**g**) live and (**b**,**d**,**f**,**h**) dead bacteria adhered on the (**a**,**b**) original SS, (**c**,**d**) SS-PDA, (**e**,**f**) SS-PDA-Br, (**g**,**h**) SS-HPVBC and (**i**,**j**) SS-PViologen surfaces after exposure to *Pseudomonas* sp. for 4 h. Scale bars are 50 µm.

The bactericidal and adhesive properties of the functionalized SS surfaces were evaluated using the marine Gram-negative bacterium *Pseudomonas* sp. NCIMB 2021. Figure 4 displays fluorescence micrographs of the original and functionalized SS surfaces following a 4 h immersion in a *Pseudomonas* sp.-inoculated medium. After staining with a combination dye, viable cells exhibited a green fluorescence, whereas dead cells appeared red under the fluorescence microscope. It has been reported that the original SS surface is highly susceptible to fouling by a diverse array of invasive bacteria [27,28]. A high concentration of bacterial cells exhibiting green fluorescence, either as individual cells or in small clusters (Figure 4a), was observed on the original SS surface. In contrast, only a few single cells displayed red fluorescence (Figure 4b). These findings indicate that the majority of *Pseudomonas* sp. cells on the original SS surface remained viable, with intact cell membranes, thereby confirming its pronounced susceptibility to bacterial adhesion, because the original SS has a smooth, hydrophilic surface and lack of antimicrobial properties, which facilitate the attachment of bacteria. Many viable bacterial cells were also observed on the SS-PDA and SS-PDA-Br surfaces (Figure 4c,e); however, the bacterial cell density on these surfaces was lower than that on the original SS substrates. Notably, there was a significantly higher number of dead cells on the SS-PDA-Br surface compared to both the original SS and SS-PDA surfaces. This observation suggests that the bromide moieties present on the SS-PDA-Br surface exert bactericidal effects.

The number of viable bacterial cells adhered to the SS-HPVBC surface (Figure 4g) was slightly lower compared to the original SS, SS-PDA and SS-PDA-Br surfaces, with the majority of the bacterial cells observed being dead. On the SS-PViologen surface, only several thinly distributed viable cells were observed (Figure 4i), and the number of dead bacterial cells (Figure 4j) was significantly reduced, compared to the SS-HPVBC surface. The SS-PViologen surface demonstrated superior antibacterial and antifouling properties, attributed to its highly hydrophilic nature, characterized by a contact angle of 17 ± 1°. This hydrophilicity results in low interfacial energy between the polymer and water. The significant degree of hydration increases the energetic penalty associated with water removal when biofoulants attempt to adhere, thereby minimizing bacterial attachment. The bactericidal activity of the SS-PViologen surface can be correlated with the surface concentration of positively charged pyridinium (N^+^) cations [29]. Pyridinium polycationic chains are known to disrupt the plasma membrane, leading to the release of intracellular contents [30]. The hydrophilic component of the SS-PViologen surface resists biofouling, while the quaternary ammonium groups serve as antimicrobial agents. Therefore, this study demonstrates that viologens, as pyridinium-type polymers with polycationic groups, are effective in functionalizing surface-oxidized metal substrates, resulting in excellent antibacterial and anti-adhesion properties.

**Figure 5:**
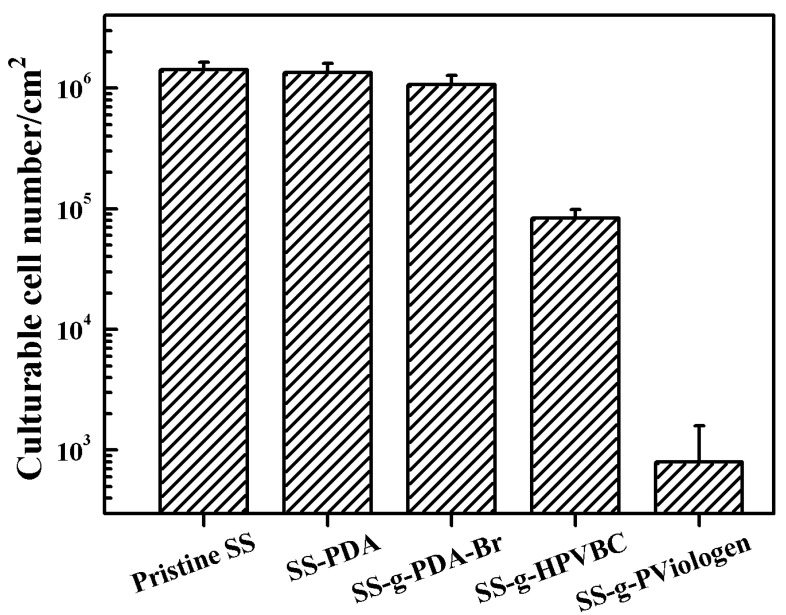
The number of culturable bacterial cells adhered per cm^2^ to both pristine and functionalized SS surfaces after a 4 h exposure to *Pseudomonas* sp. inoculated medium (10^7^ cells/mL). The cell number was determined using the spread plate method. Each error bar represents the standard deviation calculated from three replicates.

To quantify the number of culturable bacterial cells on the functionalized SS surfaces, a quantitative in vitro antibacterial assay was performed using the spread plate method. Figure 5 showed the number of culturable *Pseudomonas* sp. cells adhered to both pristine and functionalized SS surfaces. After 4 h of exposure to the inoculated medium, the culturable cell counts on the original SS, SS-PDA and SS-PDA-Br surfaces exceeded 10^6^ cells/cm^2^. In contrast, the number of adhered bacterial cells on the SS-HPVBC and SS-PViologen surfaces decreased significantly to approximately 8.5 × 10^4^ and 8 × 10^2^ cells/cm^2^, respectively, indicating a noticeable reduction in bacterial survival on the polymer-functionalized SS surfaces. The substantial decrease in culturable cells on the SS-PViologen surface further underscored the efficacy of the quaternary ammonium groups in inhibiting *Pseudomonas* sp. adhesion. These results are consistent with the qualitative trends observed in the fluorescence microscopy images.

### 2.5. Evaluation of Anti-Adhesive Properties of Functionalized SS Surfaces Against Microalgal

**Figure 6:**
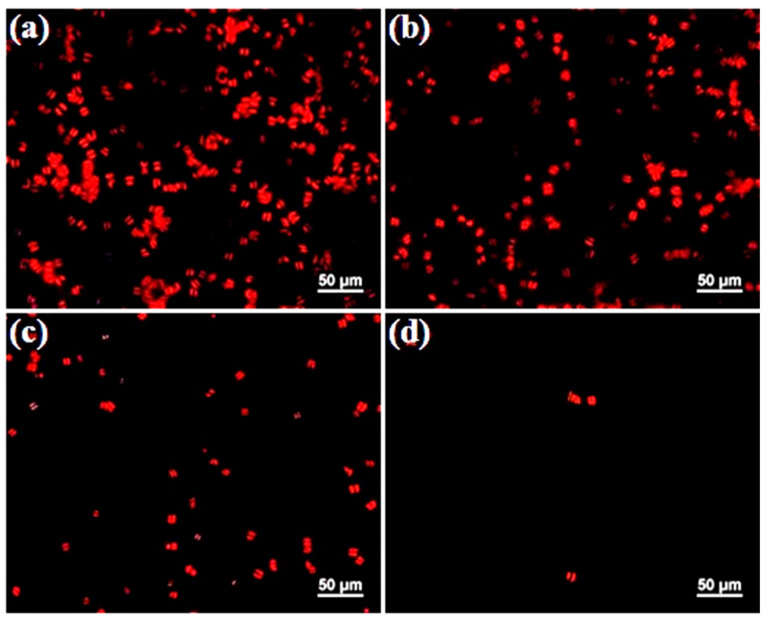
Fluorescence micrographs (1000×) of the (**a**) original SS, (**b**) SS-PDA-Br, (**c**) SS-HPVBC and (**d**) SS-PViologen surfaces after a 24 h exposure to a 30‰ salinity, filtered seawater suspension of *Amphora coffeaeformis* (density of 105 cells/mL). Scale bars are 50 µm.

*Amphora coffeaeformis*, a member of the raphid diatoms, is frequently found in abundance on fouled substrates. This species is widely recognized as a significant contributor to microfouling in the benthic zone of marine environments [31]. An *Amphora* attachment assay was conducted to assess the antifouling properties of polymer-grafted SS surfaces. The fluorescence microscopy images of *Amphora* cells adhered to the substrates after 24 h of immersion in the algal suspension are shown in Figure 6. *Amphora* heavily fouled the original SS and SS-PDA-Br surfaces (Figure 6a,b), either as individual cells or in clusters, with a slight reduction in fouling observed on the SS-HPVBC surface (Figure 6c). In contrast, only a limited number of algal cells adhered to the SS-PViologen surface (Figure 6d). The highly hydrophilic polycationic brushes on the SS-PViologen surface facilitated continuous dilution of the microalgae with water, thereby reducing their attachment to the modified surface. This observation is consistent with the low contact angle (17 ± 1°) of the SS-PViologen surface.

**Figure 7:**
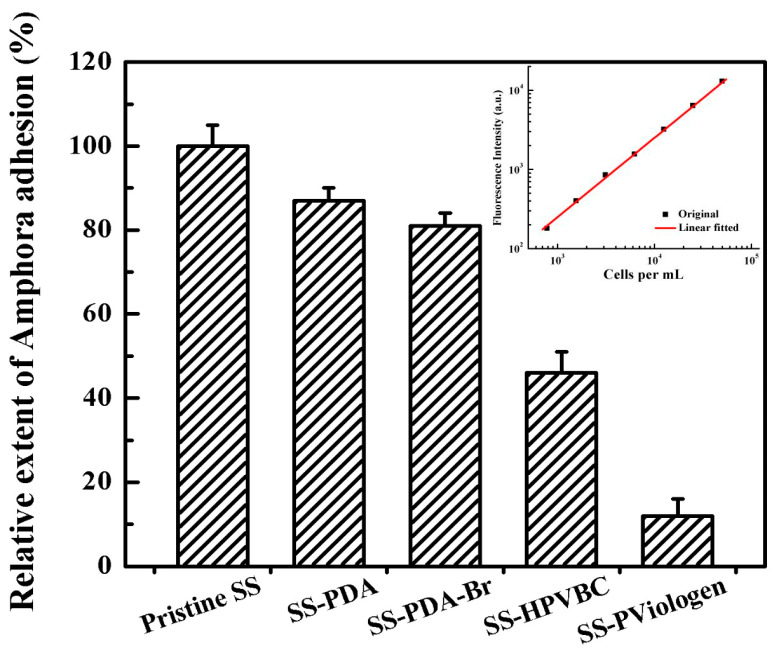
Relative extent of *Amphora* attachment on the original and functionalized SS surfaces after a 24 h exposure to the algal suspension (density of 105 cell/mL). Error bars denote the standard deviation from three replicates. The inset presents a plot depicting the relationship between the number of *Amphora* cells and their fluorescence intensity at 690 nm, along with the fitted curve.

The fluorescence micrographs offer a qualitative comparison of the effects of functionalized SS surfaces on the attachment of *Amphora coffeaeformis*. To supplement this analysis, a quantitative assessment of the adhered *Amphora* cells was conducted using a combination of ultrasonic cell removal and chlorophyll autofluorescence detection. To achieve complete detachment of the adhered algal cells from the fouled original SS surface, ultrasonic agitation was performed for 10 min, following a previously detailed procedure [20]. Under an excitation wavelength (λ_ex_) of 440 nm, chlorophyll in *Amphora coffeaeformis* cells displayed autofluorescence with a primary emission peak at approximately 690 nm. Consequently, the fluorescence intensity of the planktonic algal suspension was directly proportional to cell density, as illustrated by the calibration curve presented in the inset of Figure 7. Based on the fluorescence intensity ratios between the functionalized SS and original SS surfaces, the number of *Amphora* cells on the functionalized surfaces can be estimated. This quantification method provides a reliable means to assess the overall fouling area on a given surface.

Figure 7 shows the quantitative assay of *Amphora* attachment on the surfaces of the original SS and functionalized SS substrates. Compared to the original SS surface, the SS-PDA and SS-PDA-Br surfaces also exhibited significant fouling, with adhesion rates of 87% and 81%, respectively. In contrast, the SS-HPVBC and SS-PViologen surfaces reduced *Amphora* adhesion to 46% and 12%, respectively. These results align well with the qualitative observations made under fluorescence microscopy, with the SS-PViologen surface exhibiting the best antifouling properties. The superior antifouling properties of the SS-PViologen surface can be attributed to the highly hydrophilic quaternized viologen polymer brushes. These brushes exhibit enhanced antifouling efficacy due to their strong ability to bind water molecules, making the SS-PViologen surface more prone to interacting with water rather than biological matter, such as *Amphora* microalgal cells. Consequently, the SS-PViologen surface demonstrates the most effective resistance to *Amphora* attachment.

### 2.6. Evaluation of Anti-Settlement Properties of Functionalized SS Surfaces Against Barnacle Cyprids

**Figure 8:**
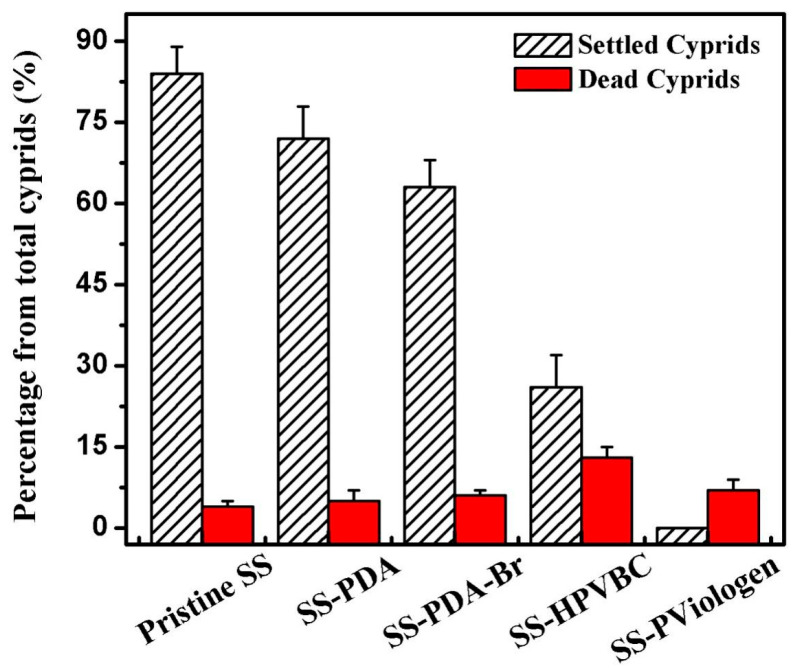
The percentage of settled and dead barnacle cyprids settled on the original and functionalized SS surfaces relative to the initial population over a 24 h settlement period. Error bars represent the standard deviation calculated from three replicates.

Barnacles are one of the most problematic and persistent macrofoulers in marine environments, exhibiting a wide geographical distribution [32,33]. In this study, Barnacles cyprids derived from *Balanus amphitrite* (adult barnacles) were selected to investigate the in vitro antifouling properties of polymer-functionalized SS surfaces under static conditions. The settlement assay was performed using the droplet method after a 24 h exposure period with a total population of approximately 40 larvae [34]. Figure 8 illustrated the percentage of settled and dead cyprids on the original and functionalized SS surfaces after 24 h of exposure. Approximately 84%, 72% and 63% of the barnacle cyprids were settled on the original SS, SS-PDA and SS-PDA-Br surfaces, respectively, indicating their high susceptibility to macrofouling. The barnacle cement at the attachment sites is a complex multi-protein composition that includes amino acid-based proteins, charged amino acid-rich proteins, highly hydrophobic proteins and an enzyme, as previously described [35]. Unlike the SS-HPVBC and SS-PViologen surfaces, the original SS, SS-PDA, and SS-PDA-Br surfaces contain a high abundance of oxygen-containing polar and functional groups, which can interact directly with proteins through ionic and hydrogen bonding [36], resulting in a higher density of barnacle cyprid settlement on these surfaces. The greatest amount of barnacle cyprid settlement on the original SS surface can be attributed to the role of the hydroxyl group in the initial priming process of underwater attachment. During this process, cement proteins are necessary for approaching the solid SS surface, where water molecules are bound. These water molecules are then displaced before the barnacle cyprids can couple with the SS surface [37].

The settlement fraction of the barnacle cyprids was significantly reduced on the polymer-grafted surfaces, with the SS-HPVBC surface exhibiting the lowest cyprid settlement rate of 26%. Despite the reduced cyprid settlement on the SS-HPVBC surface, the relatively high barnacle cyprid settlement observed can be attributed to the hydrophobic interactions between the protein molecules and the HPVBC layer. In contrast, the quaternized SS-PViologen surface showed no observable barnacle cyprid settlement (0%). The highly hydrophilic quaternized viologen polymer brushes on the SS-PViologen surface inhibit the attachment of glycoprotein cement during the settlement process of barnacle cyprids, thereby contributing to the reduced macrofouling observed on this surface. In addition to the settled barnacle cyprids, the dead fractions for the original SS, SS-PDA, SS-PDA-Br, SS-HPVBC and SS-PViologen surfaces were 4%, 5%, 6%, 13% and 7%, respectively, upon the cyprids being contact with the surfaces for 24 h. Except for the SS-HPVBC surface, the dead fractions on the functionalized SS surfaces were comparable to that of the original SS surface. The VBC-functionalized SS surface exhibited slight toxicity, but this toxicity was reduced to a non-toxic level upon coupling of the viologen moieties on the SS-HPVBC surface. With the exception of the SS-HPVBC surface, the mortality rates remained below 11%, indicating that no toxic residue was present on most of the sample substrates. Overall, the barnacle cyprid settlement assay demonstrates the excellent antifouling properties of the SS-PViologen surface.

### 2.7. Evaluation of Anti-Biocorrosion Performance of the Functionalized SS Surfaces

**Figure 9:**
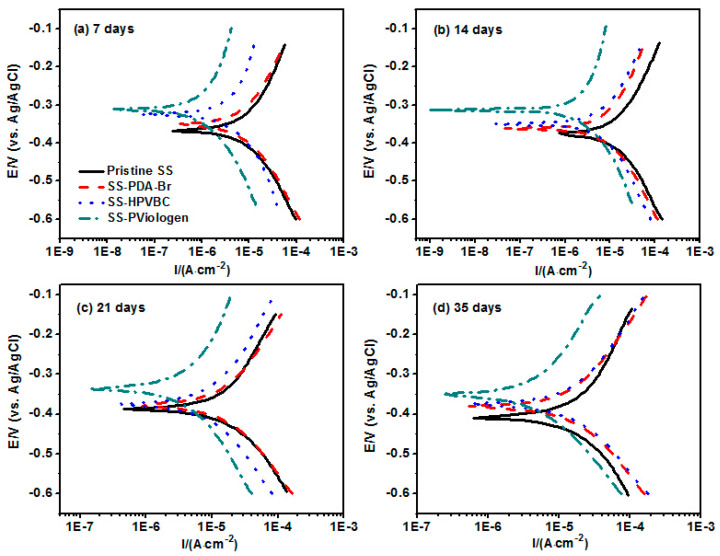
Tafel polarization curves of the original SS, SS-PDA-Br, SS-HPVBC and SS-PViologen substrates after (**a**) 7, (**b**) 14, (**c**) 21 and (**d**) 35 days of exposure to the *Pseudomonas* sp. inoculated medium.

Polarization curves are a reliable method for evaluating the instantaneous corrosion rates of metal substrates in corrosion systems containing bacteria, biocides, inhibitors, or coatings. Furthermore, this technique allows for the monitoring of electrochemical reactions on functionalized surfaces [38,39,40,41]. Figure 9 presents the Tafel plots of the original and surface-functionalized SS substrates after immersion in a *Pseudomonas* sp.-inoculated medium for 7, 14, 21 and 35 days. Tafel plots of the corresponding unexposed SS sample are shown in Electric Supplementary Information (ESI) Figure S1. The linear segments of the anodic and cathodic branches were extrapolated to their intersection to determine the Tafel slopes (β_a_ and β_c_), corrosion potentials (E_corr_) and corrosion current densities (i_corr_), as those described in detail in our previous study [42]. The fitted parameters of the Tafel polarization curves are presented in Table 2. From the Tafel polarization curves shown in Figure 9, it is evident that the E_corr_ value of the functionalized substrates exhibit slight negative shifts compared to the corresponding original SS substrates after exposure to the *Pseudomonas* sp.-inoculated medium for a predetermined period. This result is likely caused by the reduction in the cathodic reaction rate on the surface-functionalized SS substrates, as explained by the mixed potential theory.

For a given exposure period, the corrosion current density (i_corr_) value of the SS-Pviologen substrates was significantly lower than those of the corresponding original substrates, suggesting that the coupled polymer brush layers exhibited effective corrosion inhibition compared to the original SS surface in the *Pseudomonas* sp.-inoculated medium. The inhibition efficiency (IE) of the SS-PViologen surface reaches as high as circa 87.12 ± 4.36% for the unexposed samples (ESI, Figure S1), and is approximately 84.60 ± 4.23% after 7 days of exposure, and remains close to 78.20 ± 3.91% after 35 days of incubation (Table 2). The consistently high inhibition efficiency throughout the exposure period confirms that the PViologen-functionalized SS substrates provide strong corrosion protection to the underlying metallic surface, shielding it from microbial attack and other corrosive ions. It is also noteworthy that the inhibition efficiency gradually declined over time, likely due to the partial detachment of the coupled polymeric layers at specific sites on the PViologen-functionalized SS substrates.

For the original SS substrates, the corrosion rate progressively increased with exposure time in the *Pseudomonas* sp.-inoculated medium, peaking at 0.219 ± 0.0109 mm·y^−1^ after 35 days of exposure. This suggests that the oxide layer on the metal surface progressively loses its protective properties when exposed to the *Pseudomonas* sp. bacterial strain. The formation of biofilm on the original SS304 and underneath micropits upon the biofilm removal are observed on the ESI Figure S2. The significant increase in corrosion rate is ascribed to the occurrence of pitting corrosion. In contrast, the corrosion rates of the SS-PViologen surface after different exposure periods remained significantly lower than those of the original SS surfaces, demonstrating the excellent protective properties of the thin poly(viologen) layer on the SS-PViologen surface. The quaternary ammonium and pyridinium moieties within this layer play a key role in corrosion inhibition of the SS substrates. This conclusion is further supported by the substantial increase in IE value of the SS-PViologen surface compared to the original SS, SS-PDA-Br and SS-HPVBC surfaces, highlighting the superior anticorrosion properties of the SS-PViologen surface.

## 3. Materials and Methods

### 3.1. Materials

Stainless steel foils (composition of Fe/Cr18/Ni10, thickness of 0.05 mm) were procured from Goodfellow Cambridge Ltd. (Huntingdon, Cambridge, UK). Chemical reagents, including dopamine hydrochloride (98%), 2-bromoisobutyryl bromide (BIBB, 98%), *N*,*N*,*N′*,*N′*,*N″*-pentamethyldiethylenetriamine (PMDETA, 98%),4-vinylbenzyl chloride (VBC, 90%), triethylamine (TEA, 98%), 4,4′-bipyridine (98%), copper(I) bromide (CuBr, 99%) and α,α′-dichloro-p-xylene (98%) were purchased from Aladdin Scientific (Shanghai, China). These reagents were utilized as received, without further purification. The bacterial cultivation nutrients such as yeast extract, agar and peptone were sourced from Oxoid Ltd. (Hampshire, UK). *Pseudomonas* sp. NCIMB 2021, aerobic Gram-negative bacterial marine strain, was obtained from the National Collection of Marine Bacteria (Sussex, UK). The LIVE/DEAD BacLight Bacterial Viability Kit (L131152) was procured from Molecular Probes Inc. (Eugene, OR, USA). All organic solvents were of analytical grade and used as received.

### 3.2. Immobilization of Dopamine and ATRP Initiators on the SS Substrates

The SS304 foils were cut into 2 cm × 2 cm substrates and underwent ultrasonic cleaning with acetone and ethanol for 5 min each to remove grease and cleanse the surfaces. Then, the original SS substrates were thoroughly rinsed with deionized water before being dried by blowing with argon gas. The cleaned SS304 coupons were hydroxylated by immersing them in piranha solution (H_2_SO_4_ (95–97%):H_2_O_2_ (30%) = 3:1, *v*/*v*) for 30 min. This treatment efficiently eliminated native oxides and generated a hydroxyl-enriched surface, hereafter termed the SS-OH surface [43]. After hydroxylation, the SS-OH substrates were dried under reduced pressure. For dopamine immobilization, 100 mg of dopamine was added to 20 mL of Tris-HCl buffer (10 mM, pH 8.5) and stirred continuously until completely dissolved, after which the SS-OH substrates were submerged in this solution for 24 h [44]. The reaction mixture was continuously stirred to prevent nonspecific microparticle deposition on the surfaces. After the reaction, the resulting substrates were removed and subjected to thorough rinsing with deionized water before being dried in a vacuum oven. During the self-polymerization process, a polydopamine (PDA) layer formed on the SS substrates, adhering firmly to the surface and yielding the SS-PDA configuration.

To introduce alkyl bromide groups onto the substrates as ATRP initiators, the SS-PDA substrates were placed in a round-bottom flask containing 20 mL of anhydrous dichloromethane with 2.0 mL (14.4 mmol) of triethylamine (TEA). Subsequently, 1.8 mL of BIBB (3.34 g, 14.4 mmol), dissolved in 10 mL of anhydrous dichloromethane, was added dropwise to the mixture while maintaining cooling with an ice bath. The reaction proceeded at room temperature under continuous stirring for 24 h. The resulting surfaces, referred to as SS-PDA-Br, were sequentially rinsed three times with 10 mL of acetone, ethanol and deionized water before being dried in a vacuum oven at 40 °C.

### 3.3. Grafting of Vinylbenzyl Chloride via Surface-Initiated ATRP

The grafting of PHVBC onto the SS-PDA-Br surface was achieved via surface-initiated ATRP using a procedure similar to that described previously [45]. In a typical procedure, SS-PDA-Br substrates were immersed in a reaction mixture containing 1.4 mL of VBC (10 mmol), 14.4 mg of CuBr (0.1 mmol), 21 µL (0.1 mmol) of PMDETA and 30 mL of anisole. The mixture was then degassed with argon for 30 min to remove dissolved oxygen, after which the flask was rapidly sealed to initiate polymerization. The reaction proceeded at 120 °C for 24 h, yielding hyperbranched poly(vinylbenzyl chloride)-grafted substrates, designated as SS-HPVBC surfaces. After the polymerization reaction, the resulting HPVBC-grafted SS substrates were thoroughly washed with THF, methanol and deionized water to eliminate unreacted monomers and residual homopolymer. Finally, the cleaned substrates were dried in a vacuum desiccator.

### 3.4. Coupling of Viologen on the SS-HPVBC Surface

Viologen moieties were introduced onto the SS-HPVBC surface through a reaction between the SS-HPVBC substrate, α,α’-dichloro-p-xylene and 4,4′-bipyridine, as described previously [22]. Briefly, equimolar amounts of the two reactants were dissolved in DMF to prepare a 0.1 mol/L reaction mixture. The SS-HPVBC substrate was then immersed in this solution and allowed to react at 70 °C for 48 h. To further quaternize the terminal pyridine groups, a small amount of benzyl chloride was added, and the reaction continued for an additional 24 h. Following the reaction, the resulting PViologen-coupled surfaces were thoroughly washed sequentially with DMF, methanol and distilled water to remove any physically adsorbed reactants or copolymers. Finally, the viologen-functionalized SS substrates (designated as SS-PViologen) were dried overnight in a vacuum oven at 40 °C.

### 3.5. Surface Characterization

The XPS (Kratos AXIS Ultra DLD spectrometer, Kratos Analytical Inc., Manchester, UK) measurement was conducted with a monochromatic Al Kα X-ray source (1486.71 eV) to analyze the surface composition of the functionalized samples. The sessile drop method was employed to measure the static WCAs of both the original SS and functionalized SS samples at 25 °C. A 2 µL water droplet was placed on the substrates and assessed using a telescopic goniometer (Rame-Hart Inc., Succasunna, NJ, USA) that featured 23× magnification and a protractor with 1° graduation. Each reported WCA value was the mean of four substrates, with the measurement for each substrate averaged over three different surface locations.

### 3.6. Bacteria Adhesion Assay

The marine aerobic bacterium *Pseudomonas* sp. was utilized to assess bacterial adhesion properties and the bactericidal effectiveness of the PViologen-functionalized SS surface. *Pseudomonas* sp. was cultivated in artificial seawater enriched with various nutrients. The culture medium primarily consisted of salts, including NaCl (23.48 g/L), Na_2_SO_4_ (3.92 g/L), NaHCO_3_ (0.19 g/L), KCl (0.66 g/L), KBr (0.096 g/L), MgCl_2_ (5.28 g/L), CaCl_2_ (1.14 g/L) and H_3_BO_3_ (0.026 g/L), along with nutrients like bacteriological peptone (3 g/L) and yeast extract (1.5 g/L). The pH of the medium was adjusted to 7.2 ± 0.1 using a 5 M NaOH solution, and then the medium was autoclaved at 121 °C and 15 psi for 20 min for sterilization. After incubation at 37 °C for 3 days, bacterial cells were harvested from the suspension via centrifugation at 2700 rpm for 10 min using a Himac CT15RE high-speed centrifuge (Hitachi Ltd., Tokyo, Japan). The supernatant was discarded, and the bacterial pellet was washed twice with sterilized artificial seawater before resuspending to a concentration of 10^7^ cells/mL.

Each functionalized SS substrate was cut into squares with a dimension of 1 cm × 1 cm, sterilized in a 70% ethanol solution for 8 h, dried by purging with pure N_2_, and then placed in a 24-well plate. Finally, 1 mL of bacterial suspension was dropped on the surface of test samples and placed in an aerobic incubator at 37 °C for 4 h. The bacterial suspension was gently aspirated from each well using a pipette, and 1 mL of ultrapure water was carefully added along the walls of each well, followed by gentle removal of the water using pipetting. The substrates were rinsed three times with sterilized deionized water to eliminate non-adherent bacterial cells, followed by staining with the LIVE/DEAD BacLight Bacterial Viability Kit for 15 min. The adhered bacterial cells were visualized using a Nikon ECLIPSE Ti-U fluorescence microscope (Tokyo, Japan), equipped with a green filter and red filter. Bacterial adhesion and viability on the pristine and functionalized SS surfaces were quantified using the spread plate method [46]. Each test was performed in triplicate to calculate the mean value from technical replicates.

### 3.7. Amphora Coffeaeformis Attachment Assay

*Amphora coffeaeformis* (UTEX reference number B2080) was cultured in tissue culture flasks and maintained in F/2 medium at 24 °C under a 12 h light/12 h dark cycle for more than 7 days before further use [47]. *Amphora coffeaeformis* cells were harvested upon reaching confluence on the flask surface. The algal cells were carefully detached from the flask surface using a cell scraper. Aggregates of *Amphora* were broken up through continuous pipetting and filtering with Nitex 35-µm meshes. The harvested *Amphora* cell number was measured using a hemocytometer, and the cell suspension was adjusted to a concentration of 10^5^ cells/mL in the 30% salinity filtered seawater.

For the algal settlement assay, each square functionalized SS sample (dimension of 1 cm × 1 cm) was placed in 1 mL of *Amphora* suspension within a 24-well plastic plate. The settlement of *Amphora* was allowed to proceed for 24 h at 25 °C under a 12 h light/12 h dark cycle. At the predetermined settlement period, the samples were carefully rinsed three times with filtered seawater to remove loosely attached or unsettled diatoms. The rinsing procedure for the *Amphora* adhesion assay was similar to that used in the bacterial adhesion assay. For qualitative analysis, the settled *Amphora* cells on the 1 cm × 1 cm substrates were observed under a red filter using a Nikon ECLIPSE Ti-U fluorescence microscope.

To conduct further quantitative analysis, the settled diatoms were detached from each substrate by ultrasonication for 10 min in 2 mL of filtered seawater. Subsequently, 200 µL of the resultant aliquot was transferred to a 96-well microplate. Fluorescence intensity was measured at 690 nm using a Tecan Infinite M200 Microplate Reader (Männedorf, Switzerland), with an excitation wavelength (λ_ex_) set to 440 nm. The fluorescence intensity of filtered seawater served as the control [48]. Each test was carried out three times to calculate a mean value from technical replicates.

### 3.8. Barnacle Cyprids Settlement Assay

Adult barnacles (*Amphibalanus* (=*Balanus*) *amphitrite*) were generously supplied by the National University of Singapore and cultured in an open-cycle fish tank maintained at 25 °C. Brine shrimp was used to feed barnacles every day. Naupliar larvae collected from barnacles were cultured in a mixture (*v*:*v* = 1:1) of the microalgae *Tetraselmis suecica* and *Chaetoceros muelleri* with a density of 5 × 10^5^ larvae/mL [49]. The culture medium was refreshed at a two-day interval. After 5 days of incubation, the larvae metamorphosed into cyprids. The harvested cyprids were then aged for 2 days at 4 °C and acclimated to room temperature for 30 min before the settlement assay.

For the cyprid settlement assay, the droplet method was employed. Specifically, an aliquot of 500 µL of filtered seawater containing approximately 40 barnacle cyprids was dropped onto each sample (2 cm × 2 cm) of either original SS or functionalized SS substrate. The seeded sample was placed in a Petri dish, which was covered to reduce evaporation of the culture droplet. The Petri dish was then stored in airtight opaque containers lined with damp cloths to keep a dark and humid environment for 24 h. After the incubation period, the substrates were removed, and the number of settled cyprids was counted relative to their total number under a microscope. Each settlement assay was performed in triplicate to ensure reliable mean results from technical replicates.

### 3.9. Evaluation of Anti-Biocorrosion Properties of the Functionalized SS Substrates

The anti-corrosion performance of various SS substrates was assessed by measuring Tafel polarization curves using an Autolab PGSTAT electrochemical workstation (Metrohm, Herisau, Switzerland). In a typical procedure, both the original SS and functionalized SS substrates were immersed in a *Pseudomonas* sp.-inoculated medium for predetermined durations (0, 7, 14, 21 and 35 days), with the culture medium refreshed every 3 days to support growth. At each specified exposure period, the substrates were carefully withdrawn from the culture medium and embedded in poly(vinylidene difluoride) (PVDF) holders, with a circular area of 1.0 cm^2^ left exposed to function as the working electrode in a three-electrode glass corrosion cell. A platinum rod was used as the counter electrode, and an Ag/AgCl electrode served as the reference electrode. Tafel plots were recorded at a scan rate of 1 mV/s over a potential range of −250 mV to +250 mV relative to the open-circuit potential (OCP) [42]. The IE value of the functionalized SS substrates was calculated using the following formula [50]:

(1) $$IE\;\%=\frac{i_{o}-i_{corr}}{i_{o}},$$

where $i_{o}$ and $i_{corr}$ represent corrosion current densities of the original SS and functionalized SS substrates, respectively, which are determined by the analysis of the Tafel polarization curves.

To immobilize the adherent *Pseudomonas* sp. bacteria, each substrate was immersed in 1 mL of 3% (*v*/*v*) aqueous glutaraldehyde at 4 °C for 24 h, followed by serial dehydration in ethanol solutions at concentrations of 20%, 40%, 60%, 80% and 100%, with each treatment lasting 5 min. The substrates were then dried under reduced pressure and sputter-coated with a thin layer of platinum using a JFC-1300 Auto Fine Coater. SEM imaged was captured using a JSM-5600LV Model microscope (JEOL Co., Tokyo, Japan).

## 4. Conclusions

In this study, stainless steel surfaces were functionalized with hyperbranched poly(viologen) brushes as an environmentally friendly approach to inhibit biofouling and biocorrosion. Success in grafting of poly(viologen) chains onto the SS substrates was ascertained through XPS characterization and static WCA measurements. The positively charged pyridinium (N^+^) cations exhibited a strong bactericidal effect, while the hydrophilic SS-PViologen surface showed low bacterial adhesion. Additionally, the SS-PViologen surface exhibited good resistance to *Amphora coffeaeformis* (microalgae) adhesion and barnacle cyprid settlement, highlighting its outstanding antifouling functionality. These results confirmed that the effective inhibition of bacterial growth and anti-adhesion properties were achieved with the SS-PViologen surface. Furthermore, electrochemical analysis revealed that the poly(viologen)-grafted SS surface exhibited strong resistance to biocorrosion in a *Pseudomonas* sp.-inoculated medium, with a slight decrease in corrosion-inhibition efficacy after long-term exposure. Thus, the enhanced antifouling and anticorrosion properties of the SS-PViologen substrates highlight their potential as promising candidates for marine applications. However, a key limitation of this study lies in the evaluation of the functionalized SS coupons under static conditions in artificial seawater. Future work will focus on investigating the practical performance of SS-PViologen in natural aquatic environments under hydrodynamic conditions to validate its real-world applicability.

## Figures and Tables

**Table 1:** Static WCAs and chemical composition of the original SS and functionalized SS substrates.

Samples	Chemical Compositions (Molar Ratio)	Static WCAs (Mean ± SD ^c^, Degree)
Original SS	—	77 ± 3
SS-OH	—	36 ± 2
SS-PDA	[C]:[N] ^a^ = 8.7: 1.0	60 ± 3
SS-PDA-Br	[C]:[N]:[Br] ^a^ = 27.4: 1.4: 1.0	68 ± 2
SS-HPVBC	[C-H]:[C-N]:[C-Cl/C-O] ^b^ = 9.5: 1.0: 2.4	73 ± 2
SS-PViologen	[C-H]:[C-N]:[C-N^+^/C-O] ^b^ = 4.4: 1.0: 1.6	17 ± 1

^a^ Derived from the sensitivity-factor-corrected area ratio of XPS core spectrum, ^b^ Derived from the peak area ratio of the deconvoluted C 1s core XPS spectra. ^c^ SD denotes standard deviation.

**Table 2:** The analytical parameters of Tafel polarization of the original SS and functionalized SS substrates after various immersion periods in the *Pseudomonas* sp. inoculated medium.

Period (Days)	Sample Types	β_c_ ^a^ (mV·dec^−1^)	β_a_ ^b^ (mV·dec^−1^)	E_corr_ ^c^ (V)	i_corr_ (μA·cm^−2^)	CR ^d^ (mm·y^−1^)	IE ^e^ (%)
0	Original SS	−287 ± 14	251 ± 13	−0.274 ± 0.014	7.862 ± 0.393	0.0812 ± 0.0041	—
	SS-PViologen	−378 ± 19	180 ± 9	−0.216 ± 0.011	1.014 ± 0.050	0.0104 ± 0.0005	87.12 ± 4.36
7	Original SS	−253 ± 13	201 ± 10	−0.367 ± 0.018	8.083 ± 0.404	0.0873 ± 0.0043	—
	SS-PDA-Br	−200 ± 10	181 ± 9	−0.352 ± 0.018	6.273 ± 0.314	0.0677 ± 0.0034	22.39 ± 1.12
	SS-HPVBC	−430 ± 21	193 ± 10	−0.325 ± 0.016	4.364 ± 0.218	0.0471 ± 0.0024	46.01 ± 2.30
	SS-PViologen	−444 ± 22	187 ± 9	−0.311 ± 0.015	1.245 ± 0.062	0.0134 ± 0.0007	84.60 ± 4.23
14	Original SS	−198 ± 10	178 ± 9	−0.376 ± 0.019	10.640 ± 0.532	0.115 ± 0.0058	—
	SS-PDA-Br	−237 ± 12	192 ± 9	−0.362 ± 0.018	7.737 ± 0.387	0.0836 ± 0.0042	27.28 ± 1.36
	SS-HPVBC	−208 ± 11	196 ± 10	−0.35 ± 0.017	5.787 ± 0.289	0.0625 ± 0.0031	45.61 ± 2.28
	SS-PViologen	−452 ± 23	204 ± 11	−0.313 ± 0.016	2.426 ± 0.121	0.0262 ± 0.0013	77.20 ± 3.86
21	Original SS	−289 ± 15	195 ± 10	−0.386 ± 0.019	14.14 ± 0.707	0.153 ± 0.0077	—
	SS-PDA-Br	−191 ± 10	160 ± 8	−0.378 ± 0.019	10.4 ± 0.520	0.112 ± 0.0056	26.45 ± 1.32
	SS-HPVBC	−245 ± 12	209 ± 10	−0.376 ± 0.018	8.134 ± 0.416	0.0898 ± 0.0045	42.48 ± 2.12
	SS-PViologen	−296 ± 15	217 ± 11	−0.336 ± 0.017	3.583 ± 0.179	0.0387 ± 0.0019	74.66 ± 3.73
35	Original SS	−371 ± 19	303 ± 15	−0.408 ± 0.020	20.271 ± 1.014	0.219 ± 0.0109	—
	SS-PDA-Br	−247 ± 13	204 ± 10	−0.382 ± 0.019	14.024 ± 0.701	0.151 ± 0.0076	30.83 ± 1.54
	SS-HPVBC	−218 ± 11	177 ± 9	−0.376 ± 0.019	12.212 ± 0.611	0.132 ± 0.0066	39.76 ± 1.99
	SS-PViologen	−263 ± 13	191 ± 9	−0.352 ± 0.018	4.419 ± 0.221	0.0477 ± 0.0024	78.20 ± 3.91

^a^ β_c_ is the cathodic slope of the Tafel plot, ^b^ β_a_ is the anodic slope of the Tafel plot, ^c^ E_corr_ is the potential at which the current becomes zero during polarization, ^d^ CR is the corrosion rate, ^e^ IE represents the inhibition efficiency.

## Data Availability

Data are contained within the article.

## Supplementary Materials

The following supporting information can be downloaded at: https://www.mdpi.com/article/10.3390/molecules30112427/s1, Figure S1: Tafel polarization curves; Figure S2: SEM images.

## Abbreviations

The following abbreviations are used in this manuscript:

SS	Stainless steel
MIC	Microbiologically influenced corrosion
SRB	Sulfate-reducing bacteria
SI-ATRP	Surface-initiated atom transfer radical polymerization
P(DMAEMA)	Poly(2-(dimethylamino)ethyl methacrylate)
PDA	Polydopamine
VBC	4-vinylbenzyl chloride
PMDETA	*N*,*N*,*N*′,*N*′*,N*″-pentamethyldiethylenetriamine
TEA	Triethylamine
BIBB	2-bromoisobutyryl bromide
PVDF	Poly(vinylidene difluoride)
XPS	X-ray photoelectron spectroscopy
OCP	Open-circuit potential
IE	Inhibition efficiency

## References

[1] Lou Y., Chang W., Cui T., Wang J., Qian H., Ma L., Hao X., Zhang D. (2021). Microbiologically influenced corrosion inhibition mechanisms in corrosion protection: A review. Bioelectrochemistry.

[2] Rao P., Mulky L. (2023). An overview of microbiologically influenced corrosion on stainless steel. ChemBioEng Rev..

[3] Callow J.A., Callow M.E. (2011). Trends in the development of environmentally friendly fouling-resistant marine coatings. Nat. Commun..

[4] Xu X., Chang Y., Gong Y., Zhang Y., Yu Y., Peng H., Fu C. (2023). Recent advances in antifouling surface polymer brushes. ACS Appl. Polym. Mater..

[5] Yang W.J., Neoh K.G., Kang E.T., Teo S.L.M., Rittschof D. (2014). Polymer brush coatings for combating marine biofouling. Prog. Polym. Sci..

[6] Javed M.A., Neil W.C., McAdam G., Moreau J.W., Wade S.A. (2020). Microbiologically influenced corrosion of stainless steel by sulfate reducing bacteria: A tale of caution. Corrosion.

[7] Wang Y., Wharton J.A., Shenoi R.A. (2014). Ultimate strength analysis of aged steel-plated structures exposed to marine corrosion damage: A review. Corros. Sci..

[8] Wang C.G., Surat’man N.E.B., Mah J.J.Q., Qu C., Li Z. (2022). Surface antimicrobial functionalization with polymers: Fabrication, mechanisms and applications. J. Mater. Chem. B.

[9] Lv X., Wang C., Liu J., Sand W., Nabuk Etim I.-I., Zhang Y., Xu A., Duan J., Zhang R. (2024). The microbiologically influenced corrosion and protection of pipelines: A detailed review. Materials.

[10] Hou B., Yan J., Wang Y., Wu G., Guan F., Dong X., Ren Q., Pei Y., Duan J. (2022). Status and trend of microbiologically influenced corrosion and control technologies of pipelines in oil and gas field exploitation. Chem. Eng. Oil Gas.

[11] Jin H., Tian L., Bing W., Zhao J., Ren L. (2022). Bioinspired marine antifouling coatings: Status, prospects, and future. Prog. Mater. Sci..

[12] Lejars M., Margaillan A., Bressy C. (2012). Fouling release coatings: A nontoxic alternative to biocidal antifouling coatings. Chem. Rev..

[13] Zhang C., Qi Y., Guo Y., Zhang S., Xiong G., Wang K., Zhang Z. (2023). Anti-marine biofouling adhesion performance and mechanism of PDMS fouling-release coating containing PS-PEG hydrogel. Mar. Pollut. Bull..

[14] Qiu H., Feng K., Gapeeva A., Meurisch K., Kaps S., Li X., Yu L., Mishra Y.K., Adelung R., Baum M. (2022). Functional polymer materials for modern marine biofouling control. Prog. Polym. Sci..

[15] Wang B., Wang P., He B., Ma S., Liu S., Ye Q., Zhou F. (2023). Zwitterionic polymer brush-functionalized dual-crosslinked hydrogel via subsurface-initiated atom transfer radical polymerization for anti-fouling applications. Prog. Org. Coat..

[16] Yang W.J., Neoh K.G., Kang E.T., Lee S.S.C., Teo S.L.-M., Rittschof D. (2012). Functional polymer brushes via surface-initiated atom transfer radical graft polymerization for combating marine biofouling. Biofouling.

[17] Chu C.W., Higaki Y., Cheng C.H., Cheng M.H., Chang C.W., Chen J.T., Takahara A. (2017). Zwitterionic polymer brush grafting on anodic aluminum oxide membranes by surface-initiated atom transfer radical polymerization. Polym. Chem..

[18] Lainioti G.C., Druvari D. (2024). Designing Antibacterial-based quaternary ammonium coatings (surfaces) or films for biomedical applications: Recent advances. Int. J. Mol. Sci..

[19] Yuan S.J., Wan D., Liang B., Pehkonen S.O., Ting Y.P., Neoh K.G., Kang E.T. (2011). Lysozyme-coupled poly(poly(ethylene glycol) methacrylate)-stainless steel hybrids and their antifouling and antibacterial surfaces. Langmuir.

[20] Yuan S.J., Xu F.J., Pehkonen S.O., Ting Y.P., Neoh K.G., Kang E.T. (2009). Grafting of antibacterial polymers on stainless steel via surface-initiated atom transfer radical polymerization for inhibiting biocorrosion by Desulfovibrio desulfuricans. Biotechnol. Bioeng..

[21] Choi Y.S., Kang H., Kim D.G., Cha S.H., Lee J.C. (2014). Mussel-inspired dopamine-and plant-based cardanol-containing polymer coatings for multifunctional filtration membranes. ACS Appl. Mater. Interfaces.

[22] Yuan S.J., Xu F.J., Kang E.T., Pehkonen S.O. (2007). Modification of surface-oxidized copper alloy by coupling of viologens for inhibiting microbiologically influenced corrosion. J. Eelectrochem. Soc..

[23] Mishra B., Ghosh J., Dubey N.C., Tripathi B.P. (2024). Designing anti (-bio) fouling membranes with synergistic grafting of quaternized and zwitterionic polymers through surface initiated atom transfer radical polymerization. Sep. Purif. Technol..

[24] Wang N., Zhang Y., Chen J., Zhang J., Fang Q. (2017). Dopamine modified metal-organic frameworks on anti-corrosion properties of waterborne epoxy coatings. Pro. Org. Coat..

[25] Wang B., Wang P., He B., Liu S., Ye Q., Zhou F. (2022). Fabrication of ionic liquid-functionalized polystyrene nanospheres via subsurface-initiated atom transfer radical polymerization for anti-fouling application. Prog. Org. Coat..

[26] Li J., Wang M., Ye F., Li C., Zhang D. (2024). Surface-initiated atom transfer radical polymerization of triazole monomer from copper surface and its anticorrosion performance. Electrochim. Acta.

[27] Chen S., Shi J., Zhao Y., Wang W., Liao H., Liu G. (2021). Rapid fabrication of zwitterionic coating on 316L stainless steel surface for marine biofouling resistance. Prog. Org. Coat..

[28] Castañeda E., Castillo J., Pascual M., Rubio F., Vargas I., De la Iglesia R., Armijo F. (2024). Marine biocorrosion inhibition of Pseudomonas sp. biofilms on 304 stainless steel coated with poly-6-aminoindole produced by two different electrochemical methods. Prog. Org. Coat..

[29] Tiller J.C., Lee S.B., Lewis K., Klibanov A.M. (2002). Polymer surfaces derivatized with poly (vinyl-N-hexylpyridinium) kill airborne and waterborne bacteria. Biotechnol. Bioeng..

[30] Gokkaya D., Topuzogullari M., Arasoglu T., Trabzonlu K., Ozmen M.M., Abdurrahmanoğlu S. (2021). Antibacterial properties of cationic copolymers as a function of pendant alkyl chain length and degree of quaternization. Polym. Int..

[31] Tong C.Y., Derek C.J.C. (2021). Biofilm formation of benthic diatoms on commercial polyvinylidene fluoride membrane. Algal. Res..

[32] Stafslien S., Daniels J., Bahr J., Chisholm B., Ekin A., Webster D., Orihuela B., Rittschof D. (2012). An improved laboratory reattachment method for the rapid assessment of adult barnacle adhesion strength to fouling-release marine coatings. J. Coat. Technol. Res..

[33] Gil M.A., Pfaller J.B. (2016). Oceanic barnacles act as foundation species on plastic debris: Implications for marine dispersal. Sci. Rep..

[34] Kil J., Rahman R.T., Wang W., Choi S., Nam Y.S., Li S. (2023). Dual functionalized brush copolymers as versatile antifouling coatings. J. Mater. Chem. B.

[35] Kamino K., Inoue K., Maruyama T., Takamatsu N., Harayama S., Shizuri Y. (2000). Barnacle cement proteins: Importance of disulfide bonds in their insolubility. J. Biol. Chem..

[36] Yoshikawa C., Takagi R., Nakaji-Hirabayashi T., Ochi T., Kawamura Y., Thissen H. (2022). Marine antifouling coatings based on durable bottlebrush polymers. ACS Appl. Mater. Interfaces.

[37] Xiao J., Yuan X., Li W., Zhang T.C., He G., Yuan S. (2024). Cellulose-based aerogel derived N, B-co-doped porous biochar for high-performance CO_2_ capture and supercapacitor. Int. J. Biol. Macromol..

[38] Wang T., Guo Q., Zhang T.C., Zhang Y.-X., Yuan S. (2022). Large-scale prepared superhydrophobic HDTMS-modified diatomite/epoxy resin composite coatings for high-performance corrosion protection of magnesium alloys. Prog. Org. Coat..

[39] Wang Y., Wang T., Liu S., Wei Z., Zhang T.C., Yuan S. (2024). Bilayer diatomite-based composite coatings with superhydrophobic and self-healing properties for enhanced anticorrosion of AZ31B magnesium alloys. Surf. Coat. Technol..

[40] Liu X., He H., Zhang T.C., Ouyang L., Zhang Y.-X., Yuan S. (2021). Superhydrophobic and self-healing dual-function coatings based on mercaptabenzimidazole inhibitor-loaded magnesium silicate nanotubes for corrosion protection of AZ31B magnesium alloys. Chem. Eng. J..

[41] Shen W., Feng L., Liu X., Luo H., Liu Z., Tong P., Zhang W. (2016). Multiwall carbon nanotubes-reinforced epoxy hybrid coatings with high electrical conductivity and corrosion resistance prepared via electrostatic spraying. Prog. Org. Coat..

[42] Liu X., Zhang T.C., He H., Ouyang L., Yuan S. (2020). A stearic acid/CeO_2_ bilayer coating on AZ31B magnesium alloy with superhydrophobic and self-cleaning properties for corrosion inhibition. J. Alloys Compd..

[43] Zhang Y., Li M., Li B., Sheng W. (2024). Surface functionalization with polymer brushes via surface-initiated atom transfer radical polymerization: Synthesis, applications, and current challenges. Langmuir.

[44] Lee H., Dellatore S.M., Miller W.M., Messersmith P.B. (2007). Mussel-inspired surface chemistry for multifunctional coatings. Science.

[45] Cheng Z., Zhu X., Shi Z.L., Neoh K.G., Kang E.T. (2005). Polymer microspheres with permanent antibacterial surface from surface-initiated atom transfer radical polymerization. Ind. Eng. Chem. Res..

[46] Zhang B., Yan Q., Yuan S., Zhuang X.D., Zhang F. (2019). Enhanced antifouling and anticorrosion properties of stainless steel by biomimetic anchoring PEGDMA-cross-linking polycationic brushes. Ind. Eng. Chem. Res..

[47] Rasmussen K., Østgaard K. (2000). In situ autofluorescence detection of a fouling marine diatom on different surfaces. Biofouling.

[48] Gu J., Yuan S., Jiang W., Lv L., Liang B., Pehkonen S.O. (2018). Polymers for combating biocorrosion. Front. Mater..

[49] Pranantyo D., Xu L.Q., Neoh K.G., Kang E.T., Yang W., Teo S.L.M. (2014). Photoinduced anchoring and micropatterning of macroinitiators on polyurethane surfaces for graft polymerization of antifouling brush coatings. J. Mater. Chem. B.

[50] Wei Z., He H., Lin Y., Zhang T.C., Yuan S. (2025). Modification of diatomite with PEDOT and CeO_2_ to construct Superhydrophobic bilayer composite coatings for high-performance anticorrosion of magnesium alloys. Surf. Coat. Technol..

