# Supplementary material for: Functionalization of Stainless Steel with Hyperbranched Poly(viologen) Brushes for Enhanced Antimicrobial, Antifouling and Anticorrosion
Source: Molecules. 2025 May 31;30(11):2427. doi: 10.3390/molecules30112427 (PMC12156994; doi:10.3390/molecules30112427)
Supplement: Supplementary file 1 [file molecules-30-02427-s001.zip › molecules-3500603-supplementary.pdf]

Supporting Information

Functionalization of Stainless Steel with Hyperbranched Poly(Viologen) Brushes for Enhanced Antimicrobial, Antifouling and Anticorrosion

Huaqiang He [1], Youquan Liu [1], Wei Yang [1], Siqi Liu [2], Jie Wang [3], Zicheng Peng [3] and Shaojun Yuan [4,*]

[1] Research Institute of Natural Gas Technology, PetroChina Southwest Oil & Gasfield Company, Chengdu 610299, China; hehuaqiang@petrochina.com.cn (H.H.); youquan_1@petrochina.com.cn (Y.L.); yang_wei001@petrochina.com.cn (W.Y.)
[2] Research Institute of Safety, Environment Protection and Technology Supervision, PetroChina Southwest Oil & Gasfield Company, Chengdu 610299, China; liusiqi@petrochina.com.cn
[3] PetroChina Southwest Oil & Gas-Field Company, Chengdu 610051, China; wangjie16@petrochina.com.cn (J.W.); pengzch@petrochina.com.cn (Z.P.)
[4] Low-Carbon Technology & Chemical Reaction Engineering Lab, College of Chemical Engineering, Sichuan University, Chengdu 610065, China
* Correspondence: ysj@scu.edu.cn; Tel.: +86-28-8540-5201

S1. Supplementary Results

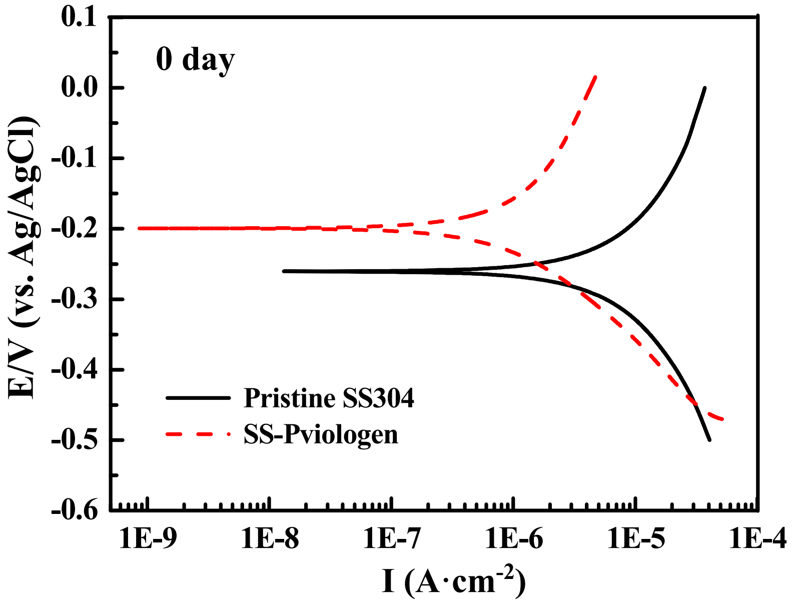

Figure S1 Tafel polarization curves of the pristine SS304 and SS-PViologen substrates before exposed to the *Pseudomonas sp.* inoculated medium (i.e., 0 day).

a

b

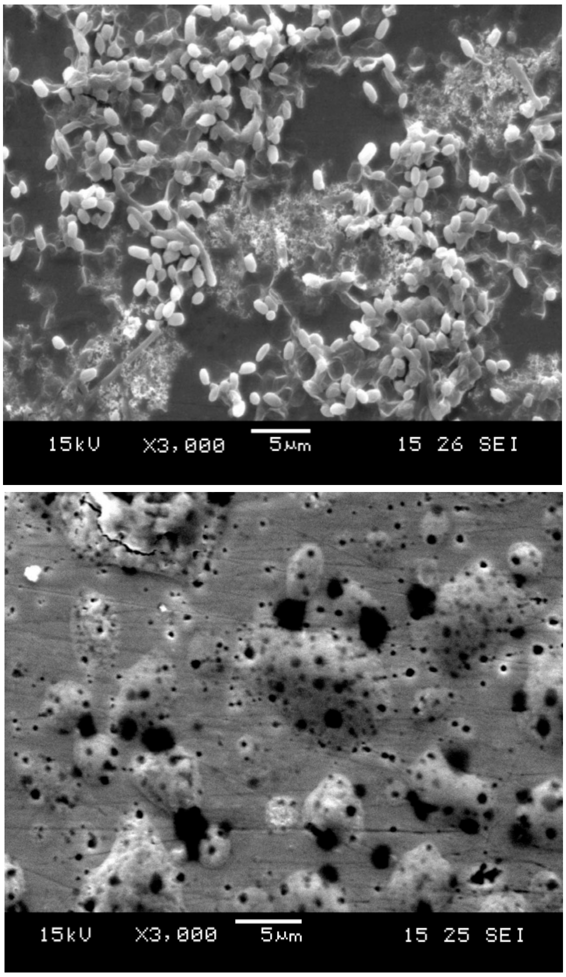

Figure S2 (a) Representative SEM images of 35 day-old biofilms formed on the 304 SS coupon surface by *Pseudomonas sp.* bacteria, (b) SEM images of the corroded coupon surfaces upon removal of the biofilms after 35 days of exposure.
